# Computed tomography derived cervical fat-free muscle fraction as an imaging-based outcome marker in patients with acute ischemic stroke: a pilot study

**DOI:** 10.1186/s12883-023-03132-7

**Published:** 2023-02-28

**Authors:** Narine Mesropyan, Louisa Khorsandian, Anton Faron, Alois M. Sprinkart, Franziska Dorn, Daniel Paech, Alexander Isaak, Daniel Kuetting, Claus C. Pieper, Alexander Radbruch, Ulrike I. Attenberger, Jens Reimann, Felix J. Bode, Cornelia Kornblum, Julian A. Luetkens

**Affiliations:** 1 Department of Diagnostic and Interventional Radiology, University Hospital Bonn, Venusberg-Campus 1, 53127 Bonn, Germany; 2 Quantitative Imaging Lab Bonn (QILaB), Venusberg-Campus 1, 53127 Bonn, Germany; 3 Department of Neurology, University Hospital Bonn, Venusberg-Campus 1, 53127 Bonn, Germany; 4 Radiologische Allianz, Andreas-Knack-Ring 16, 22307 Hamburg, Germany; 5 Department of Neuroradiology, University Hospital Bonn, Venusberg-Campus 1, 53127 Bonn, Germany

**Keywords:** Acute ischemic stroke, Computed tomography, Muscle quality, Fat-free muscle fraction

## Abstract

**Background:**

Outcome assessment in stroke patients is essential for evidence-based stroke care planning. Computed tomography (CT) is the mainstay of diagnosis in acute stroke. This study aimed to investigate whether CT-derived cervical fat-free muscle fraction (FFMF) as a biomarker of muscle quality is associated with outcome parameters after acute ischemic stroke.

**Methods:**

In this retrospective study, 66 patients (mean age: 76 ± 13 years, 30 female) with acute ischemic stroke in the anterior circulation who underwent CT, including CT-angiography, and endovascular mechanical thrombectomy of the middle cerebral artery between August 2016 and January 2020 were identified. Based on densitometric thresholds, cervical paraspinal muscles covered on CT-angiography were separated into areas of fatty and lean muscle and FFMF was calculated. The study cohort was binarized based on median FFMF (cutoff value: < 71.6%) to compare clinical variables and outcome data between two groups. Unpaired *t* test and Mann-Whitney *U* test were used for statistical analysis.

**Results:**

National Institute of Health Stroke Scale (NIHSS) (12.2 ± 4.4 vs. 13.6 ± 4.5, *P* = 0.297) and modified Rankin scale (mRS) (4.3 ± 0.9 vs. 4.4 ± 0.9, *P* = 0.475) at admission, and pre-stroke mRS (1 ± 1.3 vs. 0.9 ± 1.4, *P* = 0.489) were similar between groups with high and low FFMF. NIHSS and mRS at discharge were significantly better in patients with high FFMF compared to patients with low FFMF (NIHSS: 4.5 ± 4.4 vs. 9.5 ± 6.7; *P* = 0.004 and mRS: 2.9 ± 2.1 vs.3.9 ± 1.8; *P* = 0.049). 90-day mRS was significantly better in patients with high FFMF compared to patients with low FFMF (3.3 ± 2.2 vs. 4.3 ± 1.9, *P* = 0.045).

**Conclusion:**

Cervical FFMF obtained from routine clinical CT might be a new imaging-based muscle quality biomarker for outcome prediction in stroke patients.

## Introduction

Stroke remains the 2nd leading cause of death and the 3rd leading cause of death and disability-adjusted life years combined worldwide [1–3]. However, over the past several years these trends have begun to reverse due to advances in stroke systems of care (stoke units) providing timely imaging diagnosis followed by targeted endovascular interventions [4]. Nevertheless, stroke with its consequences remains and will remain a major public health issue. Therefore, understanding and further investigation of prognostic factors, probably leading to adverse outcome after stroke, are of great clinical importance. Different scores were developed for the assessment of global disability and outcome in stroke patients with the modified Rankin Scale (mRS) as the most used and established one [3].

Skeletal muscle quality is an established target and determinant of functional recovery and thereby outcome in patients with acute severe illness [5]. Fatty infiltration of skeletal muscle, also called myosteatosis, is a progressive multifactorial skeletal muscle affection, which is commonly associated with aging and obesity and is known to increase the risk of adverse clinical outcomes and physical disability in many pathologies [5–11]. Conventional methods for assessment of skeletal muscle quality and function, reflecting sarcopenia, such as measurement of manual grip strength or electromyography require patient cooperation, which might be difficult in the clinical setting of acute ischemic stroke [12, 13]. Skeletal muscle quality, however, may be also assessed from cervical CT data, which is routinely available from CT angiography for acute stroke diagnostic assessment. Recent studies reported that the fraction of fatty and fat-free muscle can easily be opportunistically obtained from CT and serves as a surrogate skeletal muscle quality biomarker, which can be employed for outcome assessment in acute and chronic disease [5, 11].

Therefore, the purpose of our explorative study was to investigate whether the fat-free muscle fraction (FFMF) derived from diagnostic stroke workup CT might be useful as a tool for outcome prediction in patients after acute ischemic stroke due to middle cerebral artery occlusion.

## Methods

### Study population

**Fig. 1:**
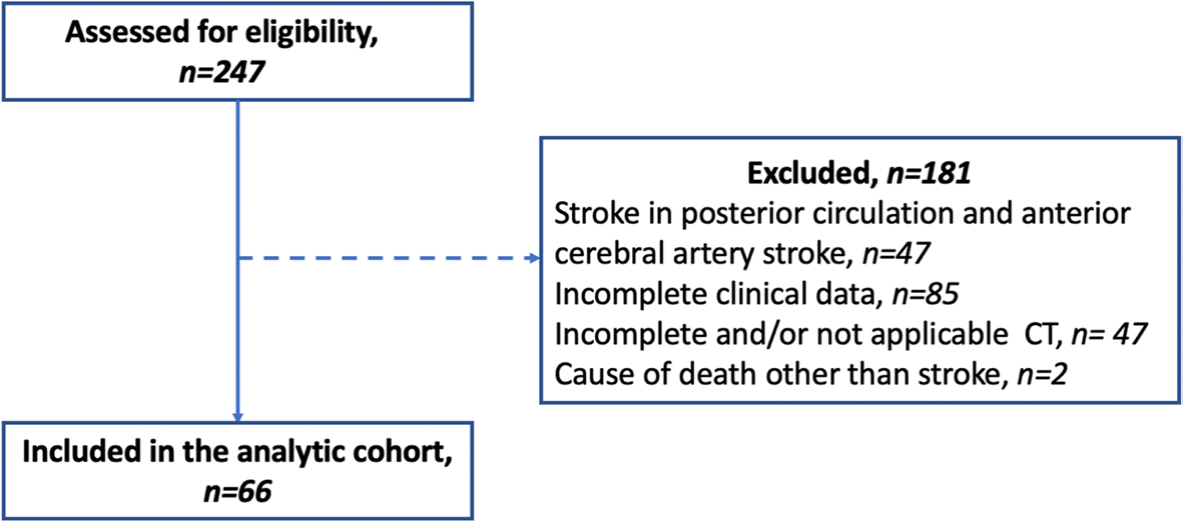
Flowchart illustrating included patients and patients which were excluded from analysis. CT: Computed tomography

This retrospective study was approved by the local institutional review board, which waived the need for patient informed consent as all examinations were performed as a part of the routine clinical workup. Between August 2016 to January 2020 patients with acute ischemic stroke from emergent large-vessel occlusion in the anterior circulation who underwent endovascular treatment with a mechanical thrombectomy were identified. Inclusion criteria were: (1) complete initial and follow-up clinical examinations and scores; (2) interpretable CT examinations, including supraaortic CT angiography; (3) acute middle cerebral artery occlusion; (4) endovascular thrombectomy of middle cerebral artery occlusion with/without prior systemic thrombolytic therapy. A flow chart demonstrating the patient recruitment process is presented in the Fig. 1. Basic demographic data (age and sex), comorbidities, preexisting risk factors for stroke, National Institutes of Health Stroke Scale (NIHSS) on admission and discharge, pre-stroke mRS, mRS on admission, discharge and after 3 month (90-day mRS), and treatment were retrieved for every patient from the electronic medical information system. Clinical evaluation on admission, discharge and after 3 months was performed by board-certified neurologists with at least 5 years of experience. The diagnosis of ischemic stroke was made based on the clinical presentation with acute neurological deficit confirmed by imaging, including vascular imaging with large-vessel occlusion of a middle cerebral artery [14]. All patients underwent diagnostic CT prior to systemic thrombolysis and endovascular intervention. The occlusion level was determined on admission CT and confirmed by digital subtraction angiography (DSA). The endovascular interventions for all patients were performed by board-certified experienced neuroradiologists on a biplane angiographic system under general anesthesia. Post endovascular revascularization success following DSA was assessed using the Thrombolysis in Cerebral Infarction (TICI) grading system.

### CT protocol

All CT examinations were performed on clinical multidetector CT scanners (Brilliance 16 CT, IQon Spectral CT, iCT 256, Philips Healthcare). CT examinations included non-enhanced CT, perfusion CT (optional) and CT angiography after injection of intravenous contrast agent (50 ml; Solutrast 370, Bracco Imaging) followed by 50 ml isotonic saline solution in bolus technique with bolus tracking in a single setting. Separate contrast media boluses in the same technique were used when both CT perfusion and CT angiography were performed. Standard parameters of image acquisition were as follows: slice thickness 1 or 2 mm, tube voltage 120 kVp, tube current (exposure time product) 82 mAs. Additionally, computer-assisted automated ASPECTS (Alberta Stroke Program Early CT Score) measurements were performed for each patient as a supportive tool for the assessment of early ischemic changes.

### CT-based skeletal muscle composition analysis

**Fig. 2:**
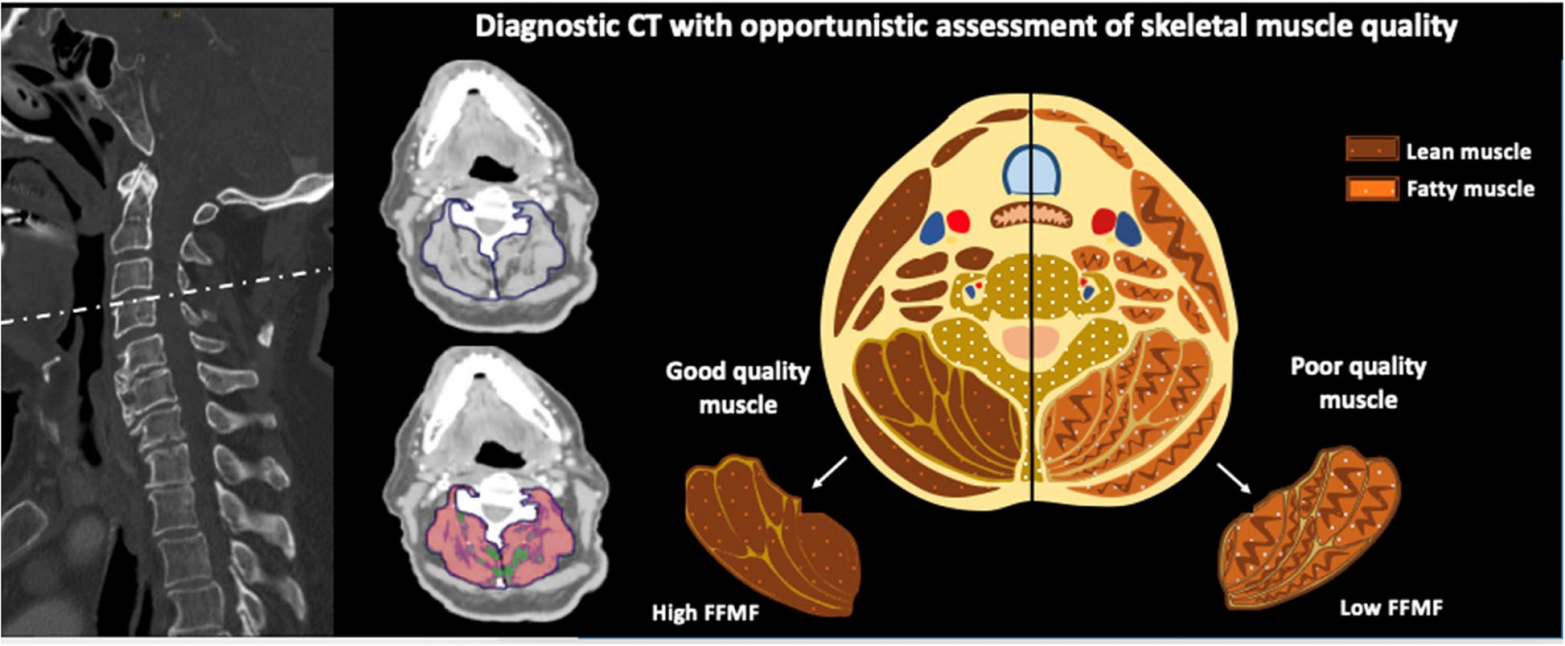
Representative images with summary of skeletal muscle composition analysis. The paraspinal muscle area at the level of the intervertebral disc space C3/4 was separated into areas of lean and fatty muscle based on densitometric thresholds. Fat-free muscle fraction (FFMF) was calculated as a relation of fatty muscle area to the total skeletal muscle area. Opportunistic analysis of CT scans was performed using an in-house software written in MATLAB. CT: Computed tomography; FFMF: Fat-free muscle fraction

For each patient, a single-slice cross-sectional image at the level of cervical intervertebral disc space C3/4 was extracted and used for opportunistic muscle composition analysis. This level was chosen because it was always covered on the supraaortic CT angiography. Image analysis was conducted by a radiology resident (N.M., with 4 years of experience in the clinical imaging) as well as a postgraduate student (L.K.) blinded to clinical data. An experienced board-certified radiologist (J.A.L., with 7 years of experience in body composition analysis) supervised the labeling process and was always available in cases of uncertainty. Muscle composition analysis was performed using an in-house software written in MATLAB as previously described [11]. For segmentation of the cervical skeletal muscle compartment, the cervical fascia was carefully traced bilaterally, separating both the left and right paraspinal skeletal muscle compartment from adjacent tissues [15, 16]. Afterwards, the total skeletal muscle area was separated into areas of fatty and lean muscle based on previously defined densitometric thresholds of − 30 to 29 Hounsfield units (HU) and 30 to 100 HU, respectively [17, 18]. Fat-free muscle fraction (FFMF) was calculated as the area of high attenuation skeletal muscle tissue referred to the total skeletal muscle area and expressed as percentage. Summary of muscle composition analysis with representative images is presented in the Fig. 2.

### Statistical analysis

Prism 9 (GraphPad Software) was used for statistical analysis. The whole study cohort was divided into two groups with patients with low and high FFMF based on the median FFMF (cutoff value: < 71.6%). Comparisons of clinical variables and outcome data was performed between these two groups. Data was checked for normal distribution using the Shapiro-Wilk test. Data is given as mean ± standard deviation or as absolute frequencies, as appropriate. Unpaired *t* test and Mann-Whitney *U* test were used for parametric and non-parametric data, respectively. Dichotomous variables were compared using x^2^ test (with a cell count greater than five) and Fischer test (with a cell count less or equal to five). Spearman correlation coefficient was used for a correlation analysis between FFMF and outcome data. Additionally, a univariable ordinal regression model was created, where FFMF is the predictor (independent) variable and the 90-day mRS is the outcome (dependent variable). In addition, power analysis using Cohen’s effect size d of the current study cohort has been performed. According to the power analysis from an effect size of d = 0.7, a reasonable power of 80% can be achieved with the available number of cases. The level of statistical significance was set on *P* < 0.05.

## Results

### Cohort characteristics

In total, 66 patients (mean age: 76 ± 13 years, 30 female) with complete clinical examination and CT data who underwent endovascular mechanical thrombectomy with or without prior systemic thrombolysis after acute ischemic stroke due to middle cerebral artery occlusion were identified (see Fig. 1). CT perfusion was performed in 6/33 (18%) and 11/33 (33%) patients with low and high FFMF, respectively. Within the study cohort 52/66 (79%) patients had a M1-segment occlusion and 14/66 (21%) patients had a M2-segment occlusion of the middle cerebral artery.

**Fig. 3:**
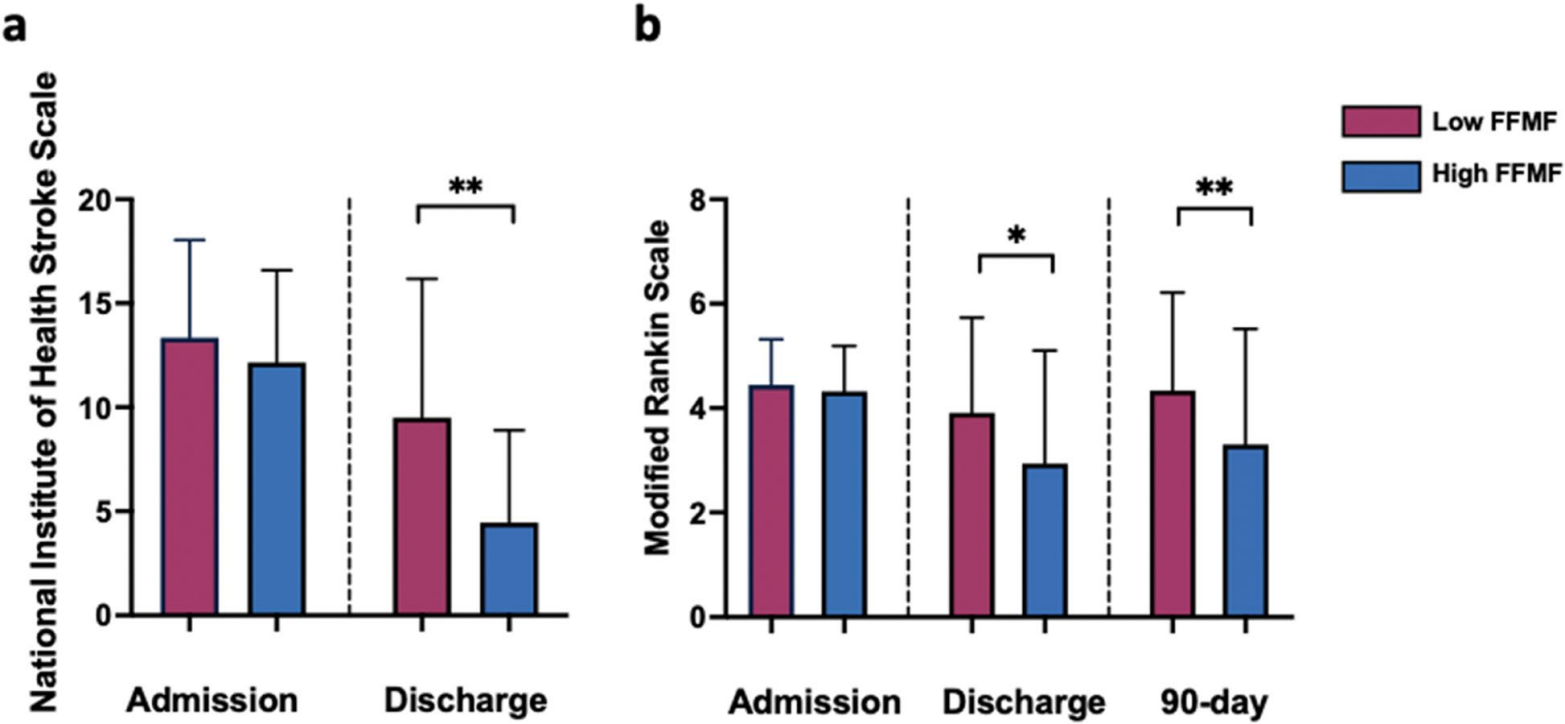
Column graphs showing the National Institute of Health Stroke (**a**) and the modified Rankin Scale (**b**) in the group of patients with low and high fat-free muscle fraction (FFMF) on admission, discharge and after 3 months. Data are presented as mean with standard deviation error bars. *, ** represents significance levels of pairwise comparisons with *P* values of ≤0.05 and ≤ 0.01, respectively. *P* values were obtained using *U* Mann-Whitney test. FFMF: Fat-free muscle fraction

Based on median FFMF the study cohort was binarized in patients with low FFMF (FFMF < 71.6%; *n* = 33) and high FFMF (≥71.6% *n* = 33). Age (*P* = 0.378) and sex (*P* = 0.999) did not differ significantly between the groups with low and high FFMF. With the only exception of atrial fibrillation (*P* < 0.001) all other possible stroke risk factors were similar between both groups (*P* > 0.05 for all comparisons). The percentage of patients with atrial fibrillation was significantly higher in the group of patients with low FFMF (73% vs. 27%, *P* < 0.001). There were no significant differences in pre-stroke mRS between patients with low and high FFMF (1 ± 1.3 vs. 0.9 ± 1.4, *P* = 0.489). On admission, there were no significant differences in neurologic deficit severity and global disability between patients with low and high FFMF as assessed by NIHSS (13.6 ± 4.5 vs. 12.2 ± 4.4; *P* = 0.297) and mRS (4.4 ± 0.9 vs. 4.3 ± 0.9; *P* = 0.475). NIHSS on discharge was significantly higher in patients with low FFMF compared to that of patients with high FFMF (9.5 ± 6.7 vs. 4.5 ± 4.4, *P* = 0.004). The mRS on discharge was significantly higher in the group of patients with a low FFMF compared to the patients with high FFMF (3.9 ± 1.8 vs. 2.9 ± 2.1, *P* = 0.049). The 90-day mRS differed also in both groups with significantly higher scale values in the group of patients with a low FFMF compared to the group of patients with a high FFMF (4.3 ± 1.9 vs. 3.3 ± 2.2, *P* = 0.045). There were 11/33 (33%) and 12/33 (36%) patients in the group with low and high FFMF, respectively, who received rehabilitation after 90 days (*P* = 0.768). All basic demographic and clinical parameters of the study cohort are presented in the Table 1, see also Fig. 3.

**Table 1:** Basic demographic, clinical and imaging characteristics of the study cohort

Variable	Low FFMF **(*n*** = 33)	High FFMF (*n* = 33)	*P* value
Age (years)	74 ± 10	71 ± 15	0.378
BMI (kg/m^2^)	26.2 ± 4.4	25.8 ± 4.7	0.368
Sex			0.999
female	15 (45%)	15 (45%)	
male	18 (55%)	18 (55%)	
TICI scale (%)			0.671
TICI 0	3 (9%)	4 (12%)	
TICI 1	–	–	
TICI 2A	1 (3%)	–	
TICI 2B	12 (36%)	9 (27%)	
TICI 3	17 (52%)	20 (61%)	
Systemic thrombolysis	15 (45%)	17 (52%)	0.806
ASPECTS	7.8 ± 1.7	7.8 ± 1.5	0.910
NIHSS on admission	13.6 ± 4.5	12.2 ± 4.4	0.297
NIHSS on discharge	9.5 ± 6.7	4.5 ± 4.4	0.004
mRS on admission	4.4 ± 0.9	4.3 ± 0.9	0.475
mRS on discharge	3.9 ± 1.8	2.9 ± 2.1	0.049
mRS at 90 days	4.3 ± 1.9	3.3 ± 2.2	0.045
Patients died	7 (21%)	7 (21%)	0.999
**Risk factors (%)**			
Arterial hypertension	30 (91%)	26 (79%)	0.303
Diabetes mellitus	7 (21%)	7 (21%)	0.999
Smoking	3 (9%)	9 (27%)	0.108
Alcohol abuse	0	0	0.999
Hypercholesterinemia	21 (64%)	19 (58%)	0.801
Previous cerebral infarction	3 (9%)	5 (15%)	0.708
Atrial fibrillation	24 (73%)	9 (27%)	< 0.001
Valvular heart disease	19 (58%)	14 (42%)	0.325
Coronary heart disease	7 (21%)	2 (6%)	0.148
**Clinical and laboratory data at admission**			
Systolic blood pressure (mmHg)	156 ± 20	154 ± 24	0.571
Diastolic blood pressure (mmHg)	86 ± 13	85 ± 14	0.726
Platelet count (G/L)	207 ± 59	254 ± 112	0.037
Pulse (times/min)	77 ± 19	83 ± 16	0.162
Creatinine (mg/dl)	1.05 ± 0.54	1.07 ± 0.89	0.943
HbA1c (%)	5.9 ± 0.7	6.3 ± 1.7	0.342
Low-density lipoprotein (mg/dl)	106 ± 33	130 ± 40	0.017
High-density lipoprotein (mg/dl)	49 ± 15	52 ± 19	0.735

*BMI* Body mass index, *FFMF* Fat-free muscle fraction, *TICI* Thrombolysis in Cerebral Infarction Scale, *NIHSS* National Institute of Health Stroke Scale, *mRS* Modified Rankin Scale, *ASPECTS* Alberta Stroke Program Early CT Score

### CT-derived parameters from skeletal muscle composition analysis

There were no significant differences in mean total skeletal muscle compartment area (40.9 ± 7.1 cm^2^ vs. 38.7 ± 8.8 cm^2^, *P* = 0.275) and corresponding skeletal muscle area (34.2 ± 5.7 cm^2^ vs. 33.9 ± 7.8 cm^2^, *P* = 0.825) between patients with low and high FFMF.

**Fig. 4:**
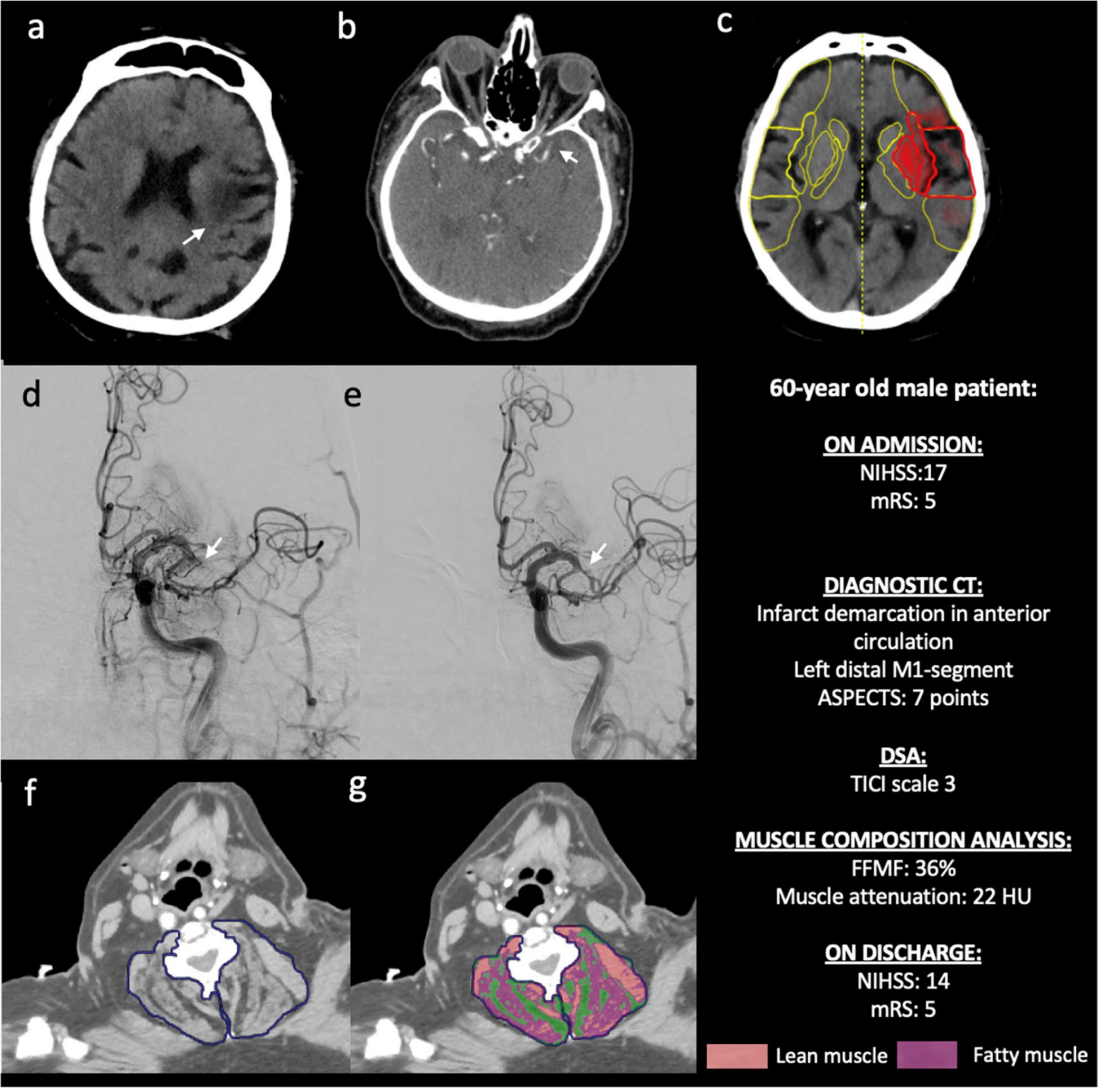
Representative images of a 60-year-old male patient with a low fat-free muscle fraction of 36% and ischemic stroke by left middle cerebral artery occlusion. Non-enhanced CT scan (**a**) demonstrates beginning infarct area demarcation (white arrow) in the anterior circulation due to occlusion of the distal M1 segment of the left middle cerebral artery (white arrow, **b**). Additional analysis of computer-assisted early ischemic changes revealed an ASPECT score of 7 (**c**). The patient underwent systemic thrombolysis and successful endovascular intervention with mechanical thrombectomy and complete recanalization (TICI scale 3) (white arrows, **d** and **e**). Images **f** and **g** visualize the assessment of skeletal muscle fat infiltration as previously described

**Fig. 5:**
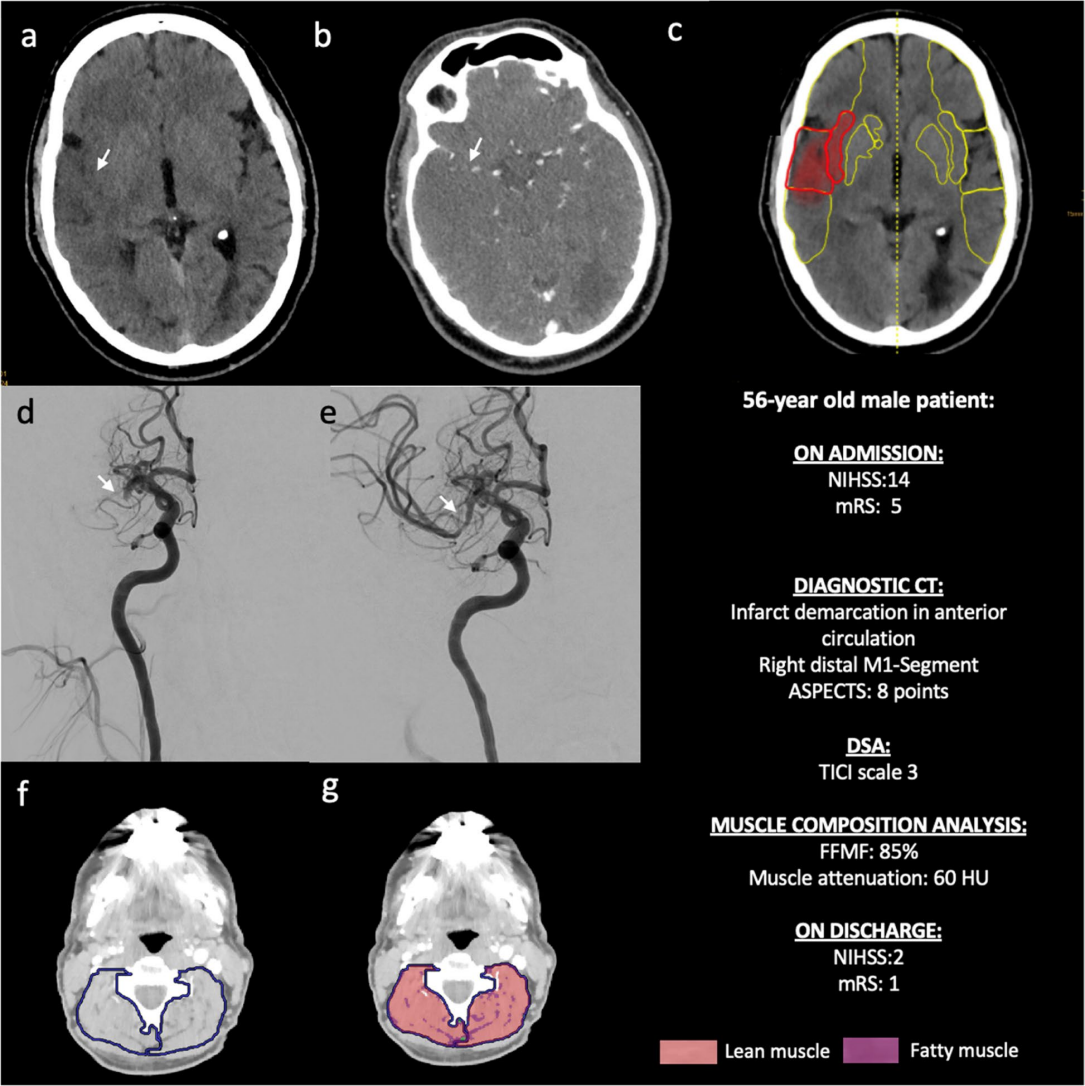
Representative images of a 56-year-old male patient with a high fat-free muscle fraction of 85% and ischemic stroke by right cerebral artery occlusion. Non-enhanced CT scan (**a**) demonstrates beginning infarct area demarcation (white arrow) in the anterior circulation due to occlusion of the distal M1 segment of the right middle cerebral artery (white arrow, **b**). Additional analysis of computer-assisted early ischemic changes revealed an ASPECT score of 8 (**c**). The patient underwent systemic thrombolysis and successful endovascular intervention with mechanical thrombectomy with complete recanalization (TICI scale 3) (white arrows, **d** and **e**). Images **f** and **g** visualize the assessment of skeletal muscle fat infiltration as previously described

Mean FFMF was 60.1 ± 10.8% in the lower median group and 79.5 ± 5.3% in the higher median group (*P* < 0.001). Mean muscle attenuation was significantly lower in patients with low compared to patients with high FFMF (32.8 ± 6.9 HU vs. 46.4 ± 6.2 HU, *P* < 0.001). The fatty muscle area in the first group was significantly higher in patients with low compared to patients with high FFMF (13.5 ± 3.8 cm^2^ vs. 6.8 ± 2.1 cm^2^, *P* < 0.001). According to the correlation analysis outcome data (NIHSS on discharge: *r* = 0.37, *P* = 0.012; mRS on discharge: *r* = 0.33, *P* = 0.007; and the 90-day mRS: *r* = 0.46, *P* = 0.032) correlated significantly with FFMF. Univariable ordinal regression analysis revealed an association between outcome data (the 90-day mRS) and CT-derived FFMF (odds ratio: 1.025; 95% confidence interval: 0.917, 1.150; *P* = 0.026). Parameters of opportunistic muscle composition analysis are presented in the Table 2. Representative clinical examples of the patients with low and high FFMF are presented in the Figs. 4 and 5.

**Table 2:** Data of CT-derived muscle composition analysis of patients with low and high fat-free muscle fraction (FFMF)

Variable	Low FFMF (*n* = 33)	High FFMF (*n* = 33)	*P* value
Total skeletal muscle compartment area (cm^2^)	40.9 ± 7.1	38.7 ± 8.8	0.275
Skeletal muscle area (cm^2^)	34.2 ± 5.7	33.9 ± 7.8	0.825
Fatty muscle area (cm^2^)	13.5 ± 3.8	6.8 ± 2.1	< 0.001
Mean muscle attenuation (HU)	32.8 ± 6.9	46.4 ± 6.2	< 0.001
Fat-free muscle fraction (%)	60.1 ± 10.8	79.5 ± 5.3	< 0.001

*FFMF* Fat-free muscle fraction, *HU* Hounsfield units

## Discussion

In this explorative study, we investigated the potential role of CT-derived FFMF as a predictor of patient outcome after emergent middle cerebral artery occlusion who underwent endovascular treatment with mechanical thrombectomy with or without prior systemic thrombolysis. Our findings suggest an association between CT-derived FFMF and patient outcome assessed by established clinical scores of neurological disabilities.

Recent advances in stroke care, particularly, targeted endovascular reperfusion therapies have reduced mortality and improved patient outcomes [2, 4, 19]. Pre-interventional imaging plays a key role in current guidelines for interventional and thrombolytic treatment decisions [19]. However, currently widely used prognostic scores for stroke outcome prediction based on clinical data may not be suitable for all cohorts due to interobserver variability, differences in racial/ethnic groups, and unknown patient background and clinical data. Therefore, more objective tools for outcome prediction, which are simply to incorporate into clinical routine are needed.

Sarcopenia is one of the main markers of muscle quality and is known to be associated with adverse health outcomes [20, 21]. However, according to current guidelines low muscle strength is a principal determinant of sarcopenia, which overtakes the role of low muscle mass [12]. Therefore, functional tests are required for the assessment of sarcopenia. Skeletal muscle fat infiltration is another key marker of muscle quality and the impact of poor muscle quality on patient outcome in different pathologies has been investigated in previous studies. In particular, CT-derived assessment of fatty muscle degeneration as a part of opportunistic body composition analysis is shown to be an independent tool for outcome prediction, e.g., in cancer survivors [22, 23], in patients undergoing transcatheter aortic valve replacement [11], after venovenous extracorporeal membrane oxygenation [5], with inflammatory bowel disease [7], coronavirus disease [8] and others [9]. There are also studies investigating myosteatosis in association with stroke, in particular after stroke (“stroke-related”) as a consequence of functional disability using different functional tests and imaging modalities [24–26]. However, relations between myosteatosis, assessed directly by cervical FFMF derived from diagnostic CT scan as a general fitness biomarker at the time of stroke has not been under investigation yet.

Our study results show that regardless of similar baseline anthropometric and demographic characteristics, pre-stroke disability, and comparable severity of global and neurological disability on admission, patients with low FFMF demonstrated a worse clinical outcome and were more likely to have persistent neurological disabilities on discharge and after 3 months. It is worth mentioning that with the only exception of atrial fibrillation (*P* < 0.001) all other major risk factors for stoke under investigation (arterial hypertension, diabetes mellitus, smoking, hypercholesterinemia, coronary heart disease, etc.) were also similar in the both groups. This might have an influence on our study results, especially in patients who underwent systemic thrombolysis prior to endovascular thrombectomy [27]. However, according to other studies no differences in outcome between large vessel occlusion stroke patients with and without atrial fibrillation were demonstrated [28]. Notably, postinterventional results after early recanalization with endovascular thrombectomy were similar in both groups (*P* = 0.671). In fact, in the group of patients with high FFMF, recanalization failed in slightly more cases (12% vs. 9%), which, however, had also no influence on general outcome in the whole group. These findings are consistent with the existing literature but in case of sarcopenia. Sarcopenia is proven to be related to adverse outcomes in stroke patients [29–32]. However, in previous studies for the assessment of sarcopenia additional measurements were performed (e.g., assessment of the muscle strength, limb and/or body skeletal muscles, functional tests, etc.), which is not always possible in an acute stroke setting.

In this study, we therefore suggest the use of cervical FFMF, representing an objective measure of lean skeletal muscle mass, as a promising new “imaging-based biomarker” in patients with acute ischemic stroke. The potentially outstanding value of FFMF is underscored by the fact that, in contrast to other frailty scores, it is quick and easy to assess from routine pre-interventional diagnostic imaging. Furthermore, there is a growing interest in opportunistic assessment of muscle composition from cross sectional imaging, also in large patient cohorts, which makes its assessment interesting for automated applications with the help of machine learning. In fact, end-to-end automated deep learning pipelines for large-scale opportunistic assessment of body composition metrics in clinical routine already exist and could be easily developed also for cervical applications [33]. In this regard, FFMF assessment would provide a quantitative marker of muscle quality, which is independent of human-related factors, anthropometric, and demographic characteristics and could be also derived from clinical CT data without additional costs.

Our study has several limitations. First, due to the retrospective nature of the study, all clinical data, including preexisting conditions, symptom onset and risk factors, were retrieved from electronic medical records and, therefore, a possible underestimation of this data due to omissions in reporting cannot be fully excluded. Second, due to the relatively small sample size we did not perform regression analyses for the outcome assessment. This should, however, be performed in upcoming larger studies. Furthermore, no follow-ups and longitudinal assessment of outcome data over a longer period was performed. Another limitation of our study is that we did not compare CT-derived cervical FFMF to that derived from other modalities for the assessment of myosteatosis (e.g., ultrasonography or MRI). However, this was beyond the scope of our explorative study and assessment of myosteatosis is no standard of care in patients with acute ischemic stroke Another limitation of our study is that we did not perform body composition analysis of other muscle regions. The loss of some other muscle types and groups (e.g., “fast twitch” anterior thigh muscles) are especially known to be associated with aging and first affected by sarcopenia (another crucial marker of muscle quality and function associated with adverse outcomes). However, whole-body CT scans for assessment of other muscles groups were not available for body composition analysis in our stroke cohort.

In conclusion, this is the first study indicating an association between CT-derived cervical FFMF and patient outcome after acute ischemic stroke. In the future, FFMF might be helpful in the assessment of body composition in the clinical setting of acute ischemic stroke, particularly as a prognostic marker for functional recovery. This study may also motivate further prospective and longitudinal studies on a larger population to investigate the role of CT-derived cervical FFMF as a potential new imaging-based biomarker for outcome prediction in stroke patients. Another possible important point for future studies could be the investigation of associations between neurological outcome scores and muscle quality markers of other muscle groups.

## Data Availability

The datasets generated during and/or analyzed during the current study are available from the corresponding author on reasonable request.

## Abbreviations

CT: Computed tomography
DSA: Digital subtraction angiography
FMF: Fatty muscle fraction
FFMF: Fat-free muscle fraction
MA: Muscle attenuation
ASPECTS: Alberta Stroke Program Early CT Score
NIHSS: National Institutes of Health Stroke Scale
mRS: Modified Rankin Scale
TICI: Thrombolysis in Cerebral Infarction

## References

[1] Feigin VL, Stark BA, Johnson CO (2021). Global, regional, and national burden of stroke and its risk factors, 1990–2019: a systematic analysis for the global burden of disease study 2019. Lancet Neurol.

[2] Navis A, Garcia-Santibanez R, Skliut M (2019). Epidemiology and outcomes of ischemic stroke and transient ischemic attack in the adult and geriatric population. J Stroke Cerebrovasc Dis.

[3] Banks JL, Marotta CA (2007). Outcomes validity and reliability of the modified Rankin scale: implications for stroke clinical trials: a literature review and synthesis. Stroke.

[4] Falk-Delgado A, Kuntze Söderqvist Å, Fransén J (2016). Improved clinical outcome 3 months after endovascular treatment, including thrombectomy, in patients with acute ischemic stroke: a meta-analysis. J Neurointerv Surg.

[5] Faron A, Kreyer S, Sprinkart AM (2020). CT fatty muscle fraction as a new parameter for muscle quality assessment predicts outcome in venovenous extracorporeal membrane oxygenation. Sci Rep.

[6] Reinders I, Murphy RA, Brouwer IA (2016). Muscle quality and Myosteatosis: novel associations with mortality risk: the age, gene/environment susceptibility (AGES)-Reykjavik study. Am J Epidemiol.

[7] O'Brien S, Kavanagh RG, Carey BW (2018). The impact of sarcopenia and myosteatosis on postoperative outcomes in patients with inflammatory bowel disease. Eur Radiol Exp.

[8] Yi X, Liu H, Zhu L, et al. Myosteatosis predicting risk of transition to severe COVID-19 infection. Clin Nutr. 2021. 10.1016/j.clnu.2021.05.031. 10.1016/j.clnu.2021.05.031 PMC8180452 34147286

[9] Morel A, Ouamri Y, Canouï-Poitrine F (2022). Myosteatosis as an independent risk factor for mortality after kidney allograft transplantation: a retrospective cohort study. J Cachexia Sarcopenia Muscle.

[10] Wang LH, Shaw DWW, Faino A (2021). Longitudinal study of MRI and functional outcome measures in facioscapulohumeral muscular dystrophy. BMC Musculoskelet Disord.

[11] Luetkens JA, Faron A, Geissler HL (2020). Opportunistic computed tomography imaging for the assessment of fatty muscle fraction predicts outcome in patients undergoing Transcatheter aortic valve replacement. Circulation.

[12] Cruz-Jentoft AJ, Bahat G, Bauer J (2019). Sarcopenia: revised European consensus on definition and diagnosis. Age Ageing.

[13] Clark DJ, Fielding RA (2012). Neuromuscular contributions to age-related weakness. J Gerontol A Biol Sci Med Sci.

[14] Sacco RL, Kasner SE, Broderick JP (2013). An updated definition of stroke for the 21st century: a statement for healthcare professionals from the American Heart Association/American Stroke Association. Stroke.

[15] Faron A, Sprinkart AM, Pieper CC (2020). Yttrium-90 radioembolization for hepatocellular carcinoma: outcome prediction with MRI derived fat-free muscle area. Eur J Radiol.

[16] Faron A, Pieper CC, Schmeel FC (2019). Fat-free muscle area measured by magnetic resonance imaging predicts overall survival of patients undergoing radioembolization of colorectal cancer liver metastases. Eur Radiol.

[17] Aubrey J, Esfandiari N, Baracos VE (2014). Measurement of skeletal muscle radiation attenuation and basis of its biological variation. Acta Physiol (Oxf).

[18] Nowak S, Faron A, Luetkens JA (2020). Fully automated segmentation of connective tissue compartments for CT-based body composition analysis: a deep learning approach. Investig Radiol.

[19] Berlet MH, Stambo GW, Kelley M (2014). Does modern ischemic stroke therapy in a large community-based dedicated stroke center improve clinical outcomes? A two-year retrospective study. J Stroke Cerebrovasc Dis.

[20] Malmstrom TK, Miller DK, Simonsick EM (2016). SARC-F: a symptom score to predict persons with sarcopenia at risk for poor functional outcomes. J Cachexia Sarcopenia Muscle.

[21] Bone AE, Hepgul N, Kon S (2017). Sarcopenia and frailty in chronic respiratory disease. Chron Respir Dis.

[22] Akahori T, Sho M, Kinoshita S (2015). Prognostic significance of muscle attenuation in pancreatic Cancer patients treated with neoadjuvant Chemoradiotherapy. World J Surg.

[23] Yamashita S, Iwahashi Y, Miyai H (2020). Myosteatosis as a novel prognostic biomarker after radical cystectomy for bladder cancer. Sci Rep.

[24] English C, McLennan H, Thoirs K (2010). Loss of skeletal muscle mass after stroke: a systematic review. Int J Stroke.

[25] Ryan AS, Dobrovolny CL, Smith GV (2002). Hemiparetic muscle atrophy and increased intramuscular fat in stroke patients. Arch Phys Med Rehabil.

[26] D'Souza A, Bolsterlee B, Herbert RD. Intramuscular fat in the medial gastrocnemius muscle of people who have had a stroke. Front Bioeng Biotechnol. 2020;8. 10.3389/fbioe.2020.00613. 10.3389/fbioe.2020.00613 PMC7296139 32582684

[27] Saposnik G, Gladstone D, Raptis R (2013). Atrial fibrillation in ischemic stroke: predicting response to thrombolysis and clinical outcomes. Stroke.

[28] Smaal JA, De Ridder IR, Heshmatollah A (2020). Effect of atrial fibrillation on endovascular thrombectomy for acute ischemic stroke. A meta-analysis of individual patient data from six randomised trials: results from the HERMES collaboration. Eur Stroke J.

[29] Su Y, Yuki M, Otsuki M (2020). Prevalence of stroke-related sarcopenia: a systematic review and meta-analysis. J Stroke Cerebrovasc Dis.

[30] Matsushita T, Nishioka S, Taguchi S, et al. Effect of improvement in sarcopenia on functional and discharge outcomes in stroke rehabilitation patients. Nutrients. 2021;13. 10.3390/nu13072192. 10.3390/nu13072192 PMC8308200 34202303

[31] Ohyama K, Watanabe M, Nosaki Y (2020). Correlation between skeletal muscle mass deficit and poor functional outcome in patients with acute ischemic stroke. J Stroke Cerebrovasc Dis.

[32] Su IJ, Li Y, Chen L (2021). The association between sarcopenia and the physical function of patients with stroke: a systematic review and meta-analysis. J Rehab Therapy.

[33] Nowak S, Theis M, Wichtmann BD, et al. End-to-end automated body composition analyses with integrated quality control for opportunistic assessment of sarcopenia in CT. Eur Radiol. 2021. 10.1007/s00330-021-08313-x. 10.1007/s00330-021-08313-x PMC9038788 34595539

